# Fibrinogen – A Practical and Cost Efficient Biomarker for Detecting Periprosthetic Joint Infection

**DOI:** 10.1038/s41598-018-27198-3

**Published:** 2018-06-11

**Authors:** S. M. Klim, F. Amerstorfer, G. Gruber, G. A. Bernhardt, R. Radl, L. Leitner, A. Leithner, M. Glehr

**Affiliations:** Department of Orthopaedics and Trauma, Medical University of Graz, Auenbruggerplatz, 5-8036 Graz, Austria

**Keywords:** Glycobiology, Diagnostic markers, Chronic pain

## Abstract

The early and accurate diagnosis of periprosthetic joint infection (PJI) can be challenging. Fibrinogen plays an important role in mediating inflammation of bacterial infections and therefore could be a valuable biomarker for PJI. The purpose of this study was to investigate the sensitivity and specificity of serum levels of fibrinogen in detecting PJI, and to compare the results with the established PJI biomarkers C-reactive protein (CRP) and leukocyte count. Eighty-four patients (124 surgeries) were prospectively included. The preoperatively analyzed parameters were fibrinogen, CRP and leukocyte count. The sensitivity and specificity of the biomarkers were calculated and compared. Fibrinogen (p < 0.001), CRP (p < 0.001) and leukocyte count (p < 0.001) had a statistically significant correlation with the criteria defining the presence of PJI. For fibrinogen, the value of 519 mg/dl had a sensitivity of 0.90 and a specificity of 0.66. The CRP cut-off point of 11.00 mg/dl had a sensitivity of 0.90 and a specificity of 0.74. The leukocyte count of 5.68 G/l had a sensitivity of 0.90 and a specificity of 0.39. Our results indicated that fibrinogen is a significant biomarker for detecting a bacterial PJI. It has shown to be a cost-efficient diagnostic support with high sensitivity and specificity.

## Introduction

Periprosthetic joint infection (PJI) is one of the most common problems in arthroplasty surgery with an incidence of 1–4% after primary total knee arthroplasty (TKA) and 1–2% after primary total hip arthroplasty (THA)^1–3^. Septic loosening causes 14.8% of all revision total knee arthroplasties (RTKA) and 9.8% of all revision total hip arthroplasties (RTHA)^4^. PJI can lead to a devastating outcome if not diagnosed properly and in a timely manner. An early diagnosis is critical, considering that the success of treatment options such as debridement, antibiotics and irrigation depends to a large extent on the time of diagnosis^5^.

To date, no single set of diagnostic criteria for PJI has been widely accepted and adopted - the diagnosis of PJI remains difficult^1,6,7^. One of the most widely accepted set of criteria used to define PJI was presented by the Musculoskeletal Infection Society (MSIS) in 2011 and modified by the International Consensus Group (ICG) on PJI in 2014^6,8^. Even in the absence of profound diagnostic criteria, PJI may still be present in the form of a low-grade and/or chronic encapsulated infection^7^. This situation may result in less intensive systemic reactions and sometimes come with normal laboratory infection markers^9^. It is fundamental for the surgeon to be able to distinguish between septic and aseptic failure, as treatment protocols and their impact on patients health differ greatly^10^. The acute diagnostic decision whether a joint arthroplasty inflammation is caused by septic or aseptic reasons is even more difficult, since it relies mainly on the clinical picture (rubor, tumor, dolor, calor) and on routinely available biomarkers such as C-reactive protein (CRP) or leukocyte count levels^11,12^.

Despite the undoubted diagnostic value of routine biomarkers like CRP and leukocyte count levels, there can be false positive results in a variety of underlying diseases such as metabolic syndrome and chronic inflammatory disease, in smokers as well as up to 30 to 60 days after a surgical procedure^13–16^. Another problem is the lack of sensitivity of CRP in detecting PJI, especially in low-grade and chronic cases^17^.

The existence of close connections between the coagulation cascade and (bacterial) infection/inflammation mechanisms has repeatedly been shown in literature^18,19^. The use of key regulators in coagulation as infection biomarkers might be a possible benefit from this knowledge. Fibrinogen, the precursor of fibrin is a soluble glycoprotein weighing 340-kDa^20^. It consists of three polypeptide chains called Aα, Bβ, and γ and is synthesized by hepatocytes with corresponding plasma levels of 150–400 mg/dl^19^. While its pivotal role in the coagulation cascade is basic knowledge, fibrinogen also plays a key role in activating and mediating the inflammation process^18,19,21,22^. This is done, among other things, by inducing and promoting the synthesis of proinflammatory cytokines such as Interleukin-6 (IL-6) and TNF-alpha in peripheral blood mononuclear cells^21^. Furthermore, fibrinogen can bind to and activate a wide range of immune cells through distinct ligand–receptor interactions^23^. Considering the mechanisms mentioned above, and knowing that fibrinogen is routinely analyzed preoperatively for coagulation analysis, without causing further costs, it could prove to be a valuable biomarker in PJI diagnosis.

This study was undertaken to (1) investigate the sensitivity and specificity of serum levels of fibrinogen in detecting PJI, and to (2) compare the sensitivity and specificity with the established PJI biomarkers CRP and leukocyte count.

## Material and Methods

**Figure 1:**
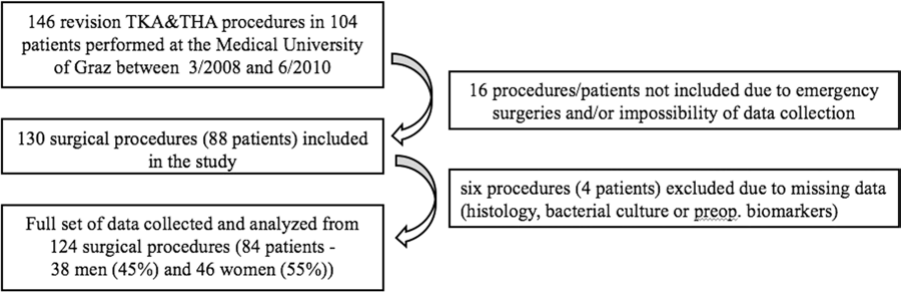
Recruitment of study patients.

Patients were prospectively recruited (Fig. 1) within 27 months at our department using the following inclusion criteria: All patients scheduled to have revision surgery after an arthroplasty of the hip or knee. Reasons for surgery were acute or chronic infection of knee and hip athroplasties or aseptic loosening of an implant. To rule out interference with other possible preconditions associated with elevated inflammatory markers, we excluded patients with inflammation other than orthopaedic infection, viral infections, rheumatic diseases, obesity (BMI > 30), heavy smoking or malignancies. Patients with renal or hepatic failure were also excluded. This study and the experimental protocols were approved by the Ethics Committee of the Medical University of Graz. The methods were carried out in accordance with relevant guidelines and regulations. Informed consent was obtained from all participants.

To determine the biomarkers of interest, blood was taken from the cubital vein on the day before surgery. Fibrinogen was analyzed by coagulometry with sodium citrate blood (normal range 210–400 mg/dl). CRP was analyzed using lithium-heparin blood and immune turbidimetry (normal < 5.0 mg/dl). Leukocytes were analysed by flow cytometry with EDTA plasma (normal range 4.4–11.3 G/l). The parameters used to define whether a PJI was present or not were either fistulation of the prosthesis or a pathogen isolated by culture from at least two separate samples obtained from the affected prosthetic joint, or three of the following six criteria: elevated ESR and CRP, elevated synovial leukocyte count, presence of purulence in the affected joint, elevated synovial neutrophil percentage, isolated microorganism in one culture, or more than five neutrophils per high-power field in five high-power fields, based on a previously published definition^24^.

The samples used for histological examination were continuously taken from the same defined localization. In knee prosthesis, synovial membrane and pseudocapsule samples were taken from the medial parapatellar membrane in knee arthroplasties and next to the femoral neck in hip arthroplasties. Vertical sections were taken from the surface of pseudocapsules facing the joint cavity. After fixating the specimens in formalin and embedding them in paraffin, they were stained with hematoxylin and eosin and examined according to the defined PJI criteria^24^. Only this technique was used in order to avoid technical histologic bias. Microbiological samples were collected before surgery (aspiration of the joint), intraoperative and/or postoperative (drainage fluid). Further, synovial membrane samples were cultured in bovine bouillon for a minimum of 10 days. After collecting all clinical and laboratory data, the patients were divided into two groups by a blinded researcher: Patients with and patients without periprosthetic joint infection.

Statistical analysis was performed using PASW software version 18 (SPSS Inc., Chicago, Illinois; 2010). We performed univariate logistic regressions and plotted receiver operating characteristic curves (ROC curves).

### Data Availability

The datasets generated during and/or analysed during the current study are available from the corresponding author on reasonable request.

## Results

Basic parameters of the two study groups are depicted in Table 1. In 16 cases (29%) of the PJI positive group, no bacteria could be isolated (after 14 days of incubation). The five cases of identified bacteria in the group without PJI were defined as non-infected and understood as reflecting contamination due to no clinical or laboratory signs of bacterial infection at the time of surgery and the six-month follow-up.

**Table 1:** Basic parameters of surgical procedures and microbiological analysis based on the data for the first surgery only (one procedure per patient).

	Group with PJI	Group without PJI
Number of patients	n = 55 (66%)	n = 29 (35%)
Number of procedures	n = 78 (62.9%)	n = 46 (37.1%)
Median age (years)	65.7 (+/−15.8)	65.1 (+/−14.6)
Affected joint	knee: n = 47 (60%) hip: n = 31 (40%)	knee: n = 21 (44.8%) hip: n = 25 (55.2%)
Median duration of surgery	82 min (20–298)	107 min (45–358)
Postop. antibiotic treatment	6 weeks	2 weeks
Bacteria identified	Staphylococci n = 30 (55%) Streptococci n = 8 (15%) Propionibacterium acnes + Enterococci n = 1 (2%) total: n = 39 (71%) of 55	Staphylococcus species n = 3 (10%) Enterobacter cloacae n = 1 (3%) Proteus mirabilis n = 1 (3%)

Of our 84 study patients, 54 were treated with a single operation, 22 with two, six with three and two with four operations. The PJI positive group was treated with explantation of the prosthesis and spacer implantation in 33 cases and in 32 cases lavage, necrectomy and exchange of all mobile parts was performed. The surgical treatment further included an exchange of the spacer in five cases as well as a Girdlestone-plastic in three cases. Further, we found a positive histology for infection in five cases, which presented no other indications for PJI. Out of these five cases, three were scheduled for reimplantation of an endoprosthesis, in one case a stem exchange and in one case an inlay exchange was performed. The PJI negative group was treated with exchange of the prosthesis in 19 cases and re-implantation of the prosthesis after spacer implantation in 16 cases due to aseptic loosening. Further procedures in this group range from an exchange of the inlay because of polyethylene wear in four cases to an arthrodesis in three cases and a Girdlestone-plastic in one case. One explantation of an endoprosthesis and two exchanges of a spacer were performed without an infection (pre-operative clinical signs of infection – elevated CRP; but post-operatively no signs of infection in histology and bacterial culture).

### Biomarker Results

**Figure 2:**
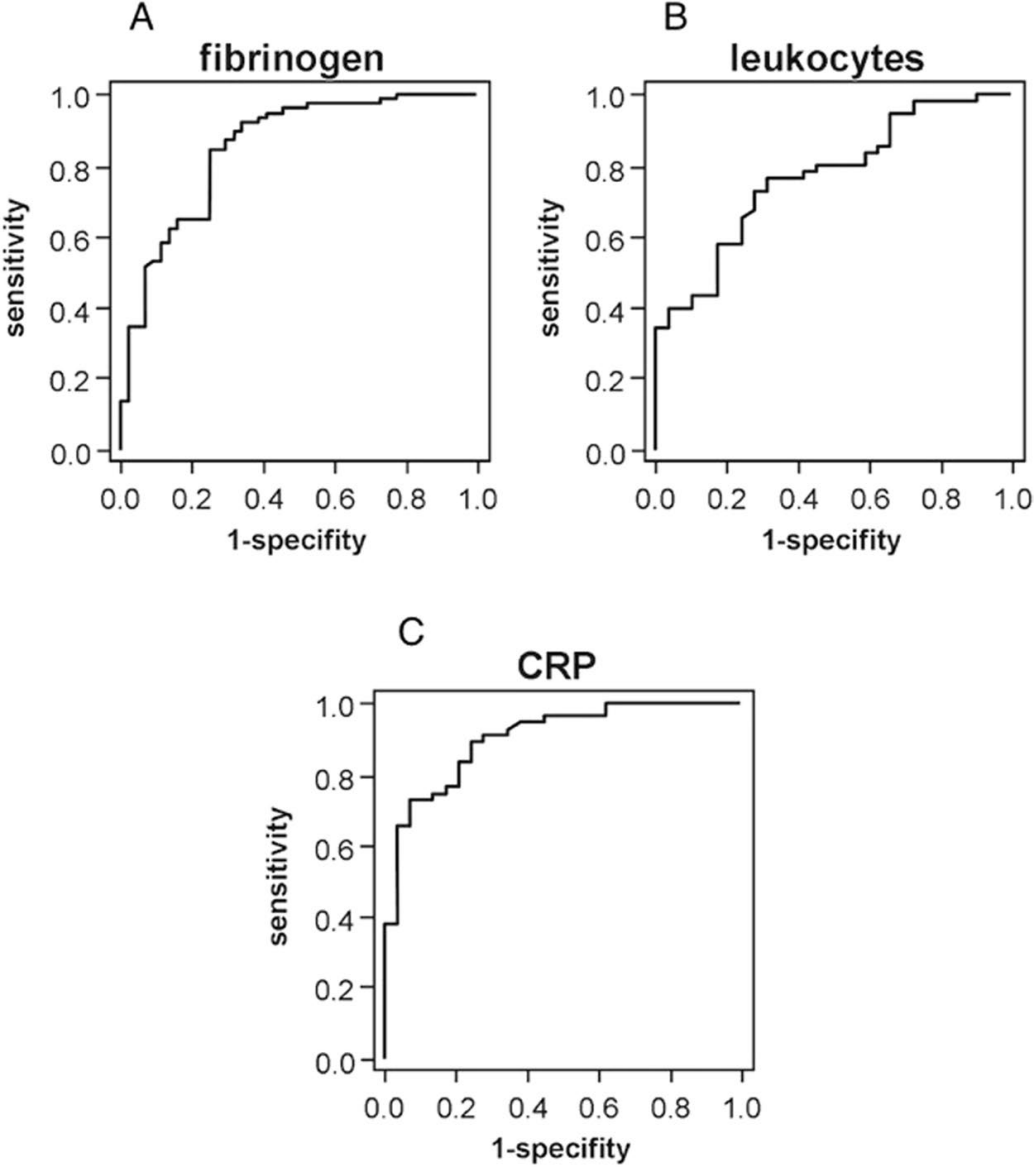
ROC curve showing the PJI predictive value of fibrinogen. (**A**) leucocytes (**B**) and CRP (**C**) for all operations. The ROC curve is a graphic plot of the positive rate (sensitivity) versus the false positive rate (specificity).

Using ROC curves, optimal thresholds were calculated for each parameter regarding sensitivity and specificity. When the data of all operations were considered, the fibrinogen value was significant (p < 0.001) for detecting bacterial infections (Fig. 2A). For fibrinogen, a value of 573.5 mg/dl had a sensitivity of 0.81 and a specificity of 0.75. The value of 519 mg/dl had a sensitivity of 0.90 and a specificity of 0.66. When only the data of the first surgeries were evaluated, the preoperative fibrinogen value was also a significant predictor for infection (p < 0.001).

CRP has proven to be significant for PJI in the first surgical procedure of each patient (p < 0.001), and when all 124 procedures were considered (p < 0.001, Fig. 2C). When only the first procedure was evaluated, the cut-off point of 23.65 mg/dl had a sensitivity of 0.8 and a specificity of 0.79, and the cut-off point of 10.25 mg/dl had a sensitivity of 0.91 and a specificity of 0.72. Counting all 124 procedures, the cut-off point of 21.95 mg/dl had a sensitivity of 0.81 and a specificity of 0.80. The cut-off point of 11.00 mg/dl had a sensitivity of 0.90 and a specificity of 0.74.

The second routine biomarker analyzed, leukocyte count, was also significant for detecting bacterial infections in both analysis groups (first procedure p = 0.001, all procedures p < 0.001; Fig. 2B). When only the first procedure was taken into account, the value of 6.27 G/l had a sensitivity of 0.8 and a specificity of 0.68, and the value of 5.48 G/l had a sensitivity of 0.91 and a specificity of 0.34. Considering all procedures, the value of 6.58 G/l had a sensitivity of 0.81 and a specificity of 0.59. The value of 5.68 G/l had a sensitivity of 0.90 and a specificity of 0.39.

## Discussion

In the literature, fibrinogen has proven to be a useful progress and/or prediction marker for a variety of inflammation-related pathologies including appendicitis^25^, periodontitis^26^, malaria^27^ and sepsis^28^. In this context, the aim of this study was to analyze the plasma concentrations of fibrinogen with respect to its sensitivity and specificity in detecting bacterial joint infections in the field of RTKA and RTHA. Additionally, we compared the results with those of the established PJI biomarkers CRP and leukocyte count.

The diagnosis of periprosthetic joint infection remains to be a challenging task even for experts in this field. Clinical signs of infection (swelling, erythema, fever, positive scintigraphy and macroscopic signs of infection during surgery such as pus) or elevated conventional biomarkers like CRP and leukocytes might be falsely positive or negative. Interpretation of positive microbiological cultures from samples taken prior to or during surgery is often difficult because infections of orthopaedic implants are frequently associated with low numbers of microorganism and it is often not possible to cultivate bacteria^29,30^. It is impossible to identify the pathogen responsible for sepsis in up to 50% of patients^31^. In our study, we could not identify a pathogen in 29% even when there were histological signs of bacterial infection. The traditional means of identifying the organism responsible for bacterial infections are non-specific (Gram stain), slow (culture), or insensitive (Gram stain and culture)^32^. Further, it is still not possible to definitely rule out a bacterial infection of a joint with the usual parameters and there is still no agreement on a gold standard for diagnosis of PJI^1,33^. Great efforts have been made by researchers trying to identify reliable biomarkers (in terms of sensitivity and specificity) to close this gap in PJI diagnosis^7,10,34–37^.

In the present study the preoperative value of serum fibrinogen was significantly higher in the PJI positive group. As part of the acute-phase reaction, fibrinogen seems to be a useful marker in the diagnostic process of joint infection. To our knowledge, there have been published only few studies examining the sensitivity and specificity of fibrinogen in detecting bacterial PJI. In the study of Alturfan *et al*.^38^ fibrinogen was a significant parameter for infection after total knee arthroplasty (432 mg/dl 93% sensitivity and 86% specificity). Sedlar *et al*.^39^ found that it was decreased after surgery and stayed at low levels up to 48 hours afterwards. Increased fibrinogen levels in plasma indicate a tendency toward hypercoagulation and in consequence entail a higher risk of thrombosis. In comparison with the results of the established PJI biomarkers CRP and leukocyte count, fibrinogen seems to be on the same level of diagnostic accuracy regarding sensitivity and specificity. This is encouraged by similar findings regarding other inflammation-related diseases^25,26^.

A set of definition criteria based on the Musculoskeletal Infection Society (MSIS) criteria was used in this study to determine the presence of PJI, as they are among the most commonly used PJI definitions with broad acceptance throughout the field^24^. However, the MSIS criteria as well as the also widely accepted Infectious Diseases Society of America (IDSA) criteria are occasionally failing to correctly detect low grade PJI^40^. Other definition criteria such as the modified Zimmerli criteria have been developed, which seem more sensitive in detecting low grade and chronic PJI^41^. However, because implant sonication analysis was not routinely performed at our hospital at time of data collection, we were not able to adopt these more stringent criteria in our study.

In the course of this study, we preoperatively examined various biomarkers for their sensitivity and specificity in detecting PJI. Among others, fibrinogen has shown to be a significant indicator^37^. Further studies are needed to evaluate the development of fibrinogen in the follow-up after septic joint surgery. Due to the study design and the unavailability of leucocyte count and differential analysis in synovial fluid in some patients at the time of data collection, we were unable to perform a comparative analysis regarding this diagnostic method. This has to be seen as a limitation, since synovial analysis has proven to be a very useful tool in detecting low grade periprosthetic joint infection. Further limitations of this study include the heterogeneity of the study population and the varying pathogenicity of the detected bacteria. Furthermore, the extent of surgical procedure differed. The different baseline values of the laboratory markers between the patients could be of bias in cases where more than one operation per patient was analyzed.

## Conclusion

The results of the present study indicate that fibrinogen is a significant biomarker for detecting an ongoing bacterial PJI. As fibrinogen is routinely analyzed for preoperative coagulation control, it causes no further costs and seems to be on the same level of accuracy with CRP and leukocytes regarding PJI detection. Although the diagnostic pathway to detect a PJI is multifactorial, fibrinogen shows to be a cost-efficient diagnostic support. Further studies will be necessary to verify our findings and identify other potential infection biomarkers in the coagulation cascade.
