# Author Correction: Fibrinogen – A Practical and Cost Efficient Biomarker for Detecting Periprosthetic Joint Infection

**DOI:** 10.1038/s41598-020-70336-z

**Published:** 2020-08-11

**Authors:** S. M. Klim, F. Amerstorfer, G. Gruber, G. A. Bernhardt, R. Radl, L. Leitner, A. Leithner, M. Glehr

**Affiliations:** Department of Orthopaedics and Trauma, Medical University of Graz, Auenbruggerplatz, 5-8036 Graz, Austria

Correction to: *Scientific Reports* https://doi.org/10.1038/s41598-018-27198-3, published online 11 June 2018

The original version of this Article contained errors as a result of an error caused during data transmission in which incorrect column conversion occurred leading to the reported mismatch in specificity outcomes.

As a result, in the Abstract,

“For fibrinogen, the value of 519 mg/dl had a sensitivity of 0.90 and a specificity of 0.34.”

now reads:

“For fibrinogen, the value of 519 mg/dl had a sensitivity of 0.90 and a specificity of 0.66.”

In the Biomarker Results section,

“For fibrinogen, a value of 573.5 mg/dl had a sensitivity of 0.81 and a specificity of 0.25. The value of 519 mg/dl had a sensitivity of 0.90 and a specificity of 0.34.”

now reads:

“For fibrinogen, a value of 573.5 mg/dl had a sensitivity of 0.81 and a specificity of 0.75. The value of 519 mg/dl had a sensitivity of 0.90 and a specificity of 0.66.”

Additionally, there was an error in the Material and Methods section where,

“We performed univariate logistic regressions and calculated non-linear mixed models with a first-order autoregressive covariance structure. Additionally, receiver operating characteristic curves (ROC curves) were plotted.”

now reads:

“We performed univariate logistic regressions and plotted receiver operating characteristic curves (ROC curves).”

These errors have now been corrected in the PDF and HTML versions of the Article.

